# The cervical anatomy of *Samotherium*, an intermediate-necked giraffid

**DOI:** 10.1098/rsos.150521

**Published:** 2015-11-25

**Authors:** Melinda Danowitz, Rebecca Domalski, Nikos Solounias

**Affiliations:** 1 Department of Anatomy, New York Institute of Technology College of Osteopathic Medicine, 8000 Northern Boulevard, Old Westbury, NY 11568, USA; 2 Department of Paleontology, American Museum of Natural History, Central Park West at 79th Street, New York, NY 10024, USA

**Keywords:** intermediate neck, Giraffidae, *Samotherium*, okapi, giraffe, cervical vertebrae

## Abstract

Giraffidae are represented by many extinct species. The only two extant taxa possess diametrically contrasting cervical morphology, as the okapi is short-necked and the giraffe is exceptionally long-necked. *Samotherium major*, known from the Late Miocene of Samos in Greece and other Eurasian localities, is a key extinct giraffid; it possesses cervical vertebrae that are intermediate in the evolutionary elongation of the neck. We describe detailed anatomical features of the cervicals of *S. major*, and compare these characteristics with the vertebrae of the two extant giraffid taxa. Based on qualitative morphological characters and a quantitative analysis of cervical dimensions, we find that the *S. major* neck is intermediate between that of the okapi and the giraffe. Specifically, the more cranial (C2–C3) vertebrae of *S. major* represent a mosaic of features shared either with the giraffe or with the okapi. The more caudal (C5–C7) *S. major* vertebrae, however, appear transitional between the two extant taxa, and hence are more unique. Notably, the C6 of *S. major* exhibits a partially excavated ventral lamina that is strong cranially but completely absent on the caudal half of the ventral vertebral body, features between those seen in the giraffe and the okapi. Comprehensive anatomical descriptions and measurements of the almost-complete cervical column reveal that *S. major* is a truly intermediate-necked giraffid. Reconstructions of the neck display our findings.

## 1. Introduction

The giraffe (*Giraffa camelopardalis*) is a spectacular mammal for its extremely long neck. The giraffe cervical vertebrae perceptibly exceed the length of necks of living and extinct ruminants. However, the only other living giraffid, the okapi (*Okapia johnstoni*), possesses relatively short cervicals, comparable to those of other ruminants [1]. The cervicals of the two extant giraffid taxa do not only differ in length; the giraffe neck exhibits homogenization of the vertebrae, whereas the okapi neck exhibits serial morphologic differences, probably relating to fighting and/or feeding [2]. Questions of the evolutionary purpose and function of the giraffe neck have been posed since Lamarck and Darwin. Many studies have used *G. camelopardalis* specimens to evaluate the physiological and anatomical considerations regarding neck lengthening. The elongation of the neck increases the distance between the giraffe heart and brain, necessitating an increase in blood pressure to compensate [3–5]. Analysis of neck lengths and masses demonstrate that the giraffe cervical vertebrae substantially elongate independently of the remainder of the vertebral column, and ultimately comprise a significant portion of total body length and mass, greater than those of coexisting ungulates [1,6].

Although remarkable, the morphological features of intermediate-necked giraffids, which play a significant role in the evolutionary transformation of the neck, remain largely unknown. While they are closely related, these species are not direct ancestors to the long-necked giraffe. Palaeotraginae are a dominant Late Miocene Eurasian giraffid subfamily that includes species of *Samotherium, Palaeotragus, Alcicephalus* and *Schansitherium* [7]. Badlangana *et al*. [1] analysed the vertebral lengths of several extinct giraffids, and determined that cervical lengthening was present among the palaeotragines [1]. A morphological study of the third cervical vertebra demonstrated two significant stages of neck elongation within Giraffidae. The palaeotragines exhibit cranial lengthening, and *Giraffa* species undergo additional caudal vertebral lengthening, ultimately leading to the elongated *G. camelopardalis* neck [8]. Detailed morphological descriptions and measurements of cervical vertebrae of the extant giraffids have been previously studied [2]. We use these anatomical comparisons to evaluate whether the neck of *Samotherium major* is truly ‘intermediate’ between the giraffe and the okapi.

Within the various species of Giraffidae, *S. major*, a species of Palaeotraginae, appears to have a neck longer than that of the okapi, but shorter than that of the giraffe. Several cervical vertebrae of this taxon, comprising almost complete necks, have been found and have been housed in museums for about 100 years. Specifically, cervical specimens from Samos in Greece can be confidently attributed to species of *S. major*. Several studies have introduced the notion that the neck of *Samotherium* spp. is intermediate, and it has been generally compared to the cervicals of young giraffes, as well as to extant ungulates, and to other extinct giraffids [1,8,9]. The anatomy and morphology of these vertebrae have never been fully described. Several vertebrae of *Samotherium sinense*, a species similar to *S. major*, have been previously described and figured [10]. A recent study of the C3 of *Samotherium* spp. gave insight to the evolutionary position of this taxon; however, a study of the entire neck is necessary because the base of the neck is functionally different from the upper vertebrae [8]. The exceptional occurrence of an almost complete neck of an intermediate giraffid allows for a comprehensive analysis of the anatomical features, and for comparisons to the short-necked okapi and long-necked giraffe. *Samotherium major* is not a direct ancestor of the giraffe or the okapi, however, it does share several common characteristics with the two extant taxa. For example, it shares with the okapi shorter metapodials and the presence of a single pair of slender ossicones, and it shares with the giraffe an anteriorly positioned soft palate and compressed bullae [9–11]. This taxon’s proposed position is a key region in the evolutionary tree of giraffids, as it represents a transitional stage of neck elongation [8,9,11]. This study provides the morphological details of the cervicals of the *S*. *major* neck, and compares characteristics with the necks of the giraffe and the okapi. In addition, the study illustrates and reconstructs the neck in anatomic position for the first time.

## 2. Material and methods

**Figure 1.**
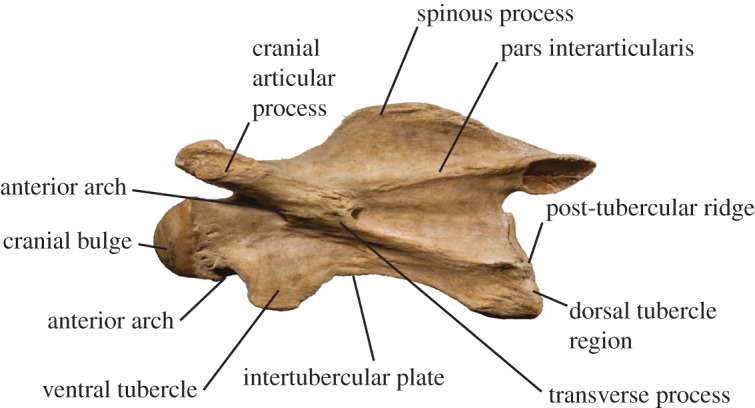
*Giraffa camelopardalis* C3 (AMNH 82001) depicting representative terminology used to describe cervical vertebrae. (See also Danowitz & Solounias [2] for vertebral terminology and descriptions).

We examine and describe the anatomical characteristics of the cervical vertebrae of *S. major*, and compare the neck morphology to that of *G. camelopardalis* and *O. johnstoni* (figure 1). The *S. major* vertebrae are housed in the Paleontological Institute of Münster (PIM) paleontology collection, and the *G. camelopardalis* and *O. johnstoni* specimens are housed in the American Museum of Natural History (AMNH) and National Museum of Natural History, Washington D.C. (NMNH) mammalogy collections. Measurements were performed on actual specimens, using standard calipers in millimetres. A description of the measurements and characters is provided in the electronic supplementary material, and a figure demonstrating the bony landmarks used can be found in Danowitz & Solounias [2]. To eliminate body size differences, each measurement is converted to a ratio to enable more accurate comparisons between the three taxa.

We perform ANOVA tests with post hoc analysis to compare cervical vertebral measurements and characters between *S. major*, *G. camelopardalis* and *O. johnstoni* using SPSS v. 22. Using a Bonferroni correction adjusting for 18 tests, statistical significance is set at the 0.0028 level. Characters in which *O. johnstoni* is not significantly different from *S. major* indicate a morphologic similarity between these taxa (likewise between *G. camelopardalis* and *S. major*). Characters in which *S. major* is not significantly different from both *O. johnstoni* and *G. camelopardalis* indicate an intermediate state between that of the two extant taxa. We subdivide the neck into two parts; these tests are performed evaluating features of the cranial (C2–C3) and caudal (C5–C7) cervical vertebrae.

### 2.1 Institutional abbreviations

AMNH, American Museum of Natural History, New York, USA. MGL, Geological Museum of Lausanne, Switzerland. NHMBa, Natural History Museum of Basel, Switzerland. NMNH, National Museum of Natural History, Washington D.C., USA. PIM, Paleontological Institute of Münster, Germany. SMNS, Stuttgart State Museum of Natural History, Germany.

## 3. Results

### 3.1 Description of *Samotherium major* cervical vertebrae

**Figure 2.**
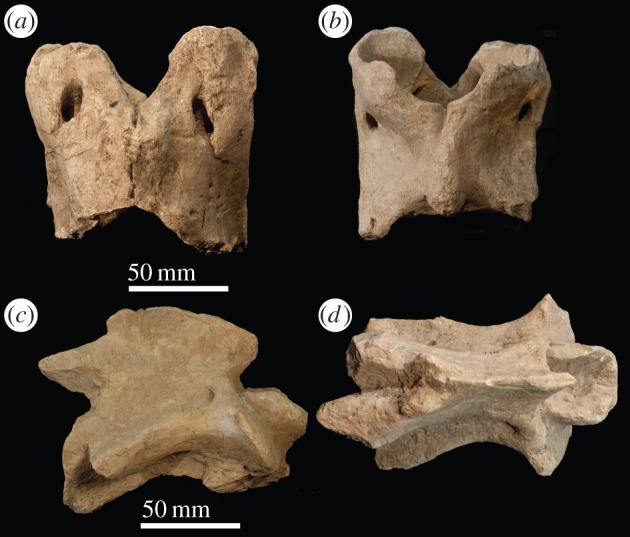
*Samotherium major* atlas (PIM 429) in (*a*) dorsal and (*b*) ventral views. *Samotherium major axis* (PIM 430) in (*c*) lateral and (*d*) dorsal views.

*Atlas* (figure 2). The vertebra comprises a dorsal and ventral arch, replacing the vertebral body, lamina and pedicle. The dorsal tubercle is positioned centrally as an expanded bony concentration on the dorsal arch, and it is shorter than the ventral tubercle. The cranial margin of the dorsal and ventral arches is U-shaped, and the ventral caudal margin forms a transverse edge. The caudal margin of the dorsal arch terminates on the same plane as that of the ventral arch. In lateral view, the alar wing is thin, sigmoid shaped and slanted dorsally. The lateral wall of the alar wings is slightly excavated, and the single midline ventral tubercle presents as a large bulge on the caudal margin of the ventral arch, which protrudes caudally. The ventral lamina exceeds the length of the dens. In cranial view, there is a concavity in the cranial vertebral foramen that corresponds to an inner-lateral notch on the articular facet for the occipital condyles.

*Axis* (figure 2). In dorsal view, the position of greatest thickness of the spinous process is central/caudal, and the dorsal articular facets are approximated towards the midline. The spinous process is arched, inclined caudally, and has a notch on the cranial edge. The cranial edge of the spinous process is positioned caudal to the cranial edge of the dens. The dens is concave on the dorsal surface, forming a trough. It has a flattened, transverse cranial end and extends cranial to the cranial most edge of the spinous process. The caudal articular facets are oval shaped. In lateral view, the dorsal tubercle protrudes from the caudal vertebral body, and connects to the transverse process with a strong, thickened ridge. The ventral ridge is continuous throughout the length of the vertebral body. It terminates caudally in a lateral expansion of bony material.

**Figure 3.**
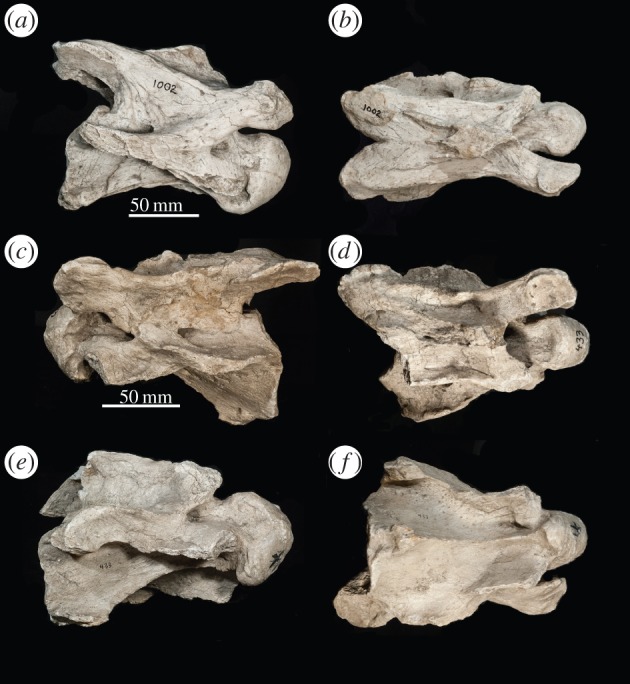
*Samotherium major* C3 vertebra (PIM 1002) in (*a*) lateral and (*b*) dorsal views. *Samotherium major* C5 vertebra (PIM 433) in (*c*) left lateral, (*d*) dorsal, (*e*) right lateral and (*f*) ventral views.

*C3* (figure 3). In dorsal view, the spinous process is situated at the base of the cranial articular processes. It is bifid with a flattened apex and is directed cranially. The spinous process extends the majority of the length of the dorsal vertebral body. Three distinct ridges radiate from the caudal aspect of the spinous process onto the dorsal lamina. The cranial articular facets are oriented dorsomedially and are rounded. The cranial articular process is elongated and tubular shaped. The cranial articular process and facet extend cranially to the same level as the tip of the cranial bulge. In lateral view, the transverse process forms a thickened, distinct, curved ridge, which presumably would connect to the dorsal tubercle. Although the dorsal tubercle is broken, the post-tubercular ridge indicates that the protrusion is situated on the caudal aspect of the vertebra. The ventral tubercle is also broken, but the base indicates that it was directed cranially. In lateral view, the caudal aspect of the vertebra is notably larger than the cranial part. In ventral view, the cranial end of the vertebra is narrower than the caudal end. The cranial bulge is domed and spherical. The cranial bulge has an elongated triangular bony expansion onto the cranial ventral vertebral body, termed the ventral extension. The ventral ridge is prominent and continuous longitudinally on the vertebral body.

*C5* (figure 3). In dorsal view, the thickest concentration of bony material on the spinous process is situated between the bases of the cranial articular processes. The spinous process extends the majority of the length of the dorsal vertebral body. Similar to C3, there are three ridges radiating from the caudal aspect of the spinous process, but these ridges are less prominent. In lateral view, the spinous process is oriented cranially and is relatively short. The dorsal lamina has a concavity lateral to the spinous process. The transverse process protrudes more laterally than the dorsal lamina and is visible in dorsal view. The cranial articular process is elongated and tubular, and the cranial articular facet is rounded. The dorsal tubercle is large, crescent-shaped and aligned with the transverse process. The dorsal tubercle is very caudal on the vertebra, and it connects to the transverse process with a curved ridge. Together, these two structures form a large, laterally flattened surface. Although the ventral tubercle is broken, it is evident from the base that it is oriented strongly cranially. A step separates the ventral tubercle from the dorsal tubercle and transverse process. In certain specimens, the cranial end of the ventral vertebral body is significantly narrower than the caudal end, and the intertubercular plate connecting the two ends is curved and sigmoid shaped. In other specimens, the ventral vertebral body is more rectangular, with the intertubercular plate oriented linearly from cranial to caudal. The caudal aspect of the intertubercular plate expands laterally. There is a fossa on the ventral vertebral body, ventral to the dorsal tubercle and transverse process. The caudal margin of the ventral vertebral body terminates in a transverse edge. The ventral extension of the cranial bulge is less elongated and prominent than in C3. The ventral ridge is less prominent than C3 but is still continuous longitudinally. At the caudal margin, the ventral ridge expands bilaterally.

**Figure 4.**
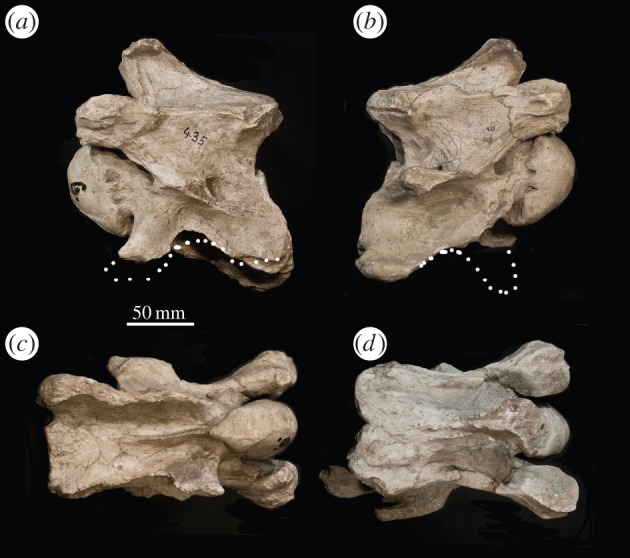
*Samotherium major* C6 vertebra (PIM 435) with reconstructed ventral lamina in (*a*) left lateral, (*b*) right lateral, (*c*) ventral and (*d*) dorsal views.

*C6* (figure 4). The thickest portion of the spinous process is situated at the base of the cranial articular processes. The base of the spinous process is more massive and expanded on the caudal aspect of the dorsal lamina. In lateral view, the spinous process is more elongated than the more proximal cervical vertebrae, and it is oriented cranially. The dorsal lamina has a concavity lateral to the spinous process. The cranial articular processes are wider and shorter than those of the more proximal cervical vertebrae, and the facets themselves remain rounded and oriented dorsomedially. There is a dorsal tubercle on the same plane as the ventral tubercle, and an accessory dorsal tubercle directly caudal to the transverse process, which presents as a curved ridge. Both the dorsal tubercle and the accessory dorsal tubercle are connected to the caudal vertebral body with an elevated post-tubercular ridge. Although portions are fragmented, the ventral tubercle appears as a distinct protrusion, rather than part of the expanded ventral lamina of a typical C6. Instead, the ventral tubercle connects to the dorsal tubercle with the intertubercular plate seen in C3–C5, and there is a notch on the lateral wall. The ventral tubercle is oriented cranially. The ventral vertebral body creates an hourglass shape, with a constriction centrally. The vertebral body is inclined cranially in relation to the entire vertebra. The ventral extension of the cranial bulge is less prominent and elongated than the more proximal cervical vertebrae. Unlike the other vertebrae, the ventral ridge is strong cranially and is completely absent on the caudal half of the ventral vertebral body. The ventral ridge is separated into two ridges with a median longitudinal groove, unique to all known ruminants.

**Figure 5.**
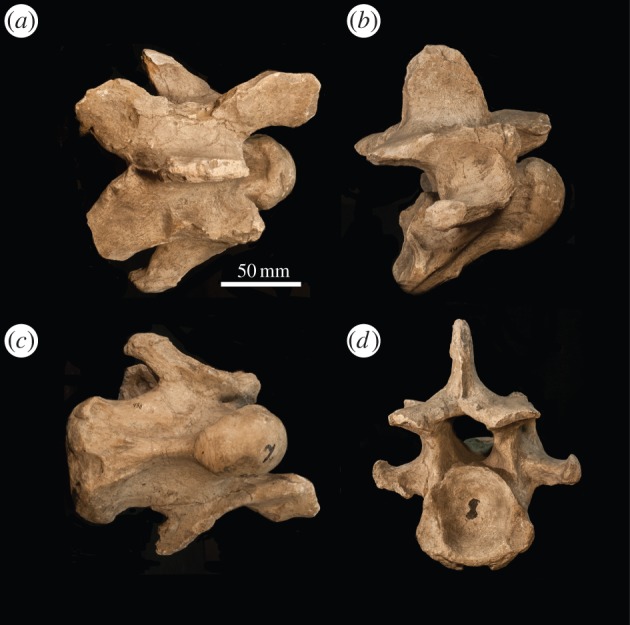
*Samotherium major* C7 vertebra (PIM 436) in (*a*) dorsal, (*b*) lateral, (*c*) ventral and (*d*) caudal views.

*C7* (figure 5). In dorsal view, the spinous process presents as a longitudinal bony thickening comprising the majority of the length of the dorsal lamina. The spinous process is shorter than that of a typical C7, and the surface is unique as it is smooth without bony protrusions or prominent striations. In lateral view, the spinous process is rounded and shaped like a parabola and is oriented dorsally. The caudal aspect of the dorsal lamina forms a widened V-shaped notch between the protruding caudal articular facets. The dorsal lamina is smooth, with faint/absent laminar ridges. The cranial articular facet is elongated and tubular, and curves medially. There is a ridge directly connecting the cranial articular facet to the transverse process. The dorsal tubercle presents as a small thickening on the caudal aspect of the transverse process, which is horizontal and very prominent. The transverse process is long and wide and is directed caudally. The vertebral body is oblong in relation to the dorsal lamina. The ventral vertebral body is boxy-shaped, but with the caudal end more laterally expanded and thickened than the rest of the vertebra. The ventral extension of the cranial bulge is expansive and rounded. Similar to C6, the ventral ridge is present and strong cranially and does not continue onto the caudal half of the ventral vertebral body. In place of the ventral ridge, there is a lateral bony expansion on the surface of the caudal vertebral body with several bony protrusions and thin elevated ridges.

### 3.2 Comparisons between *Samotherium major* and *Okapia johnstoni/Giraffa camelopardalis*

**Figure 6.**
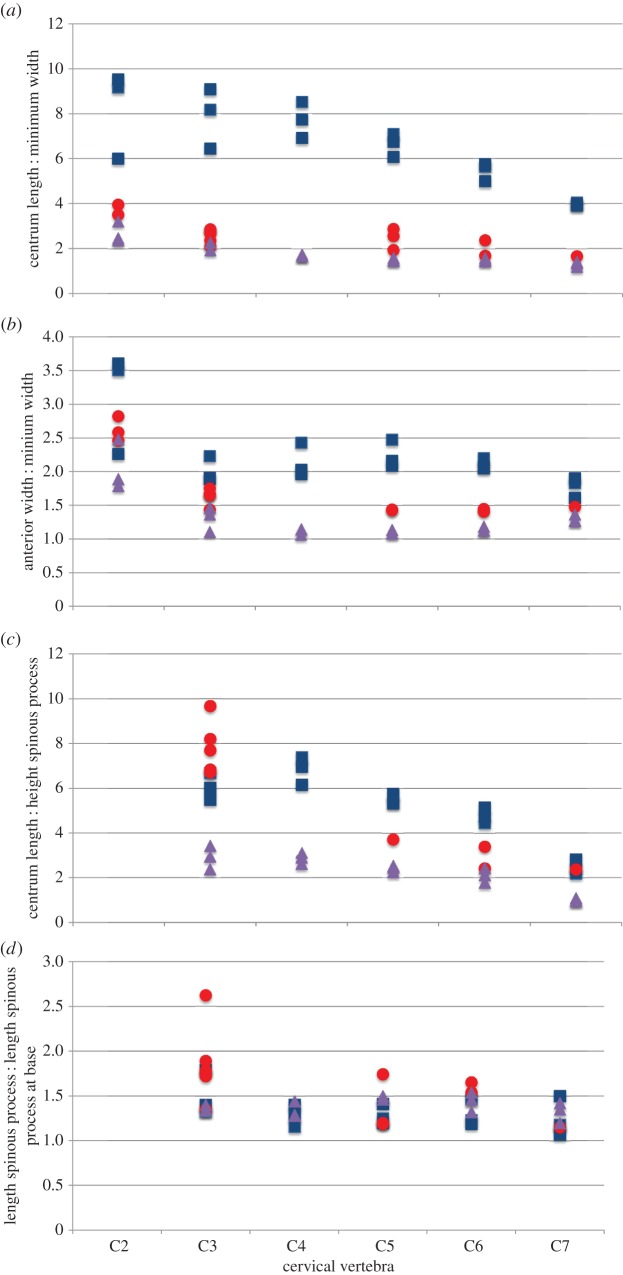

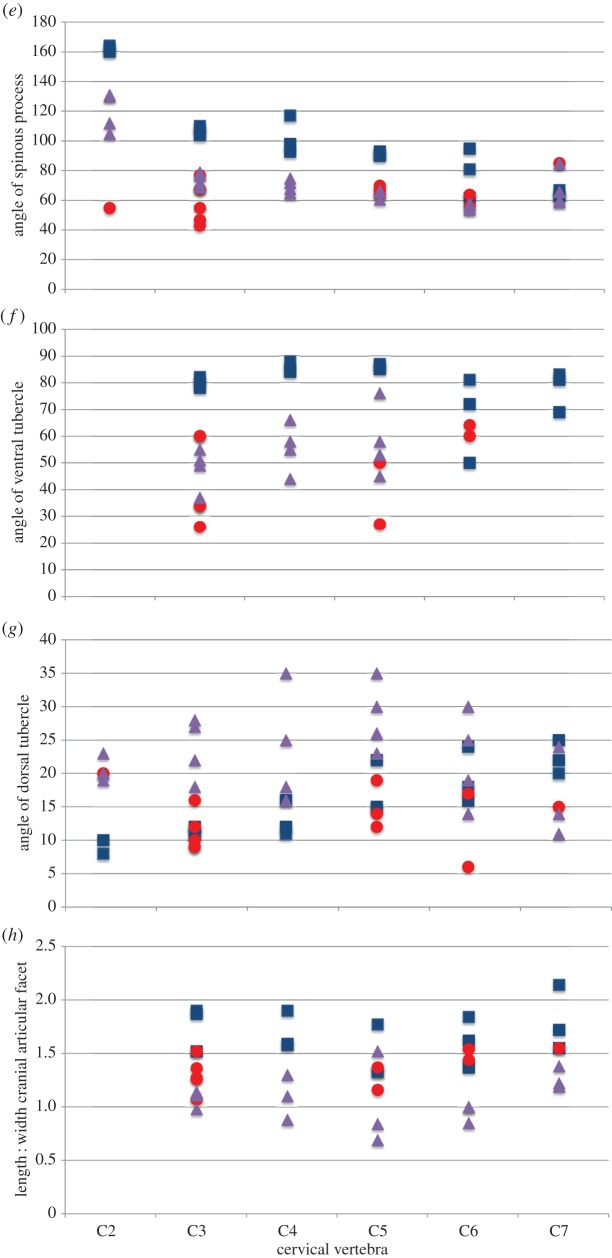

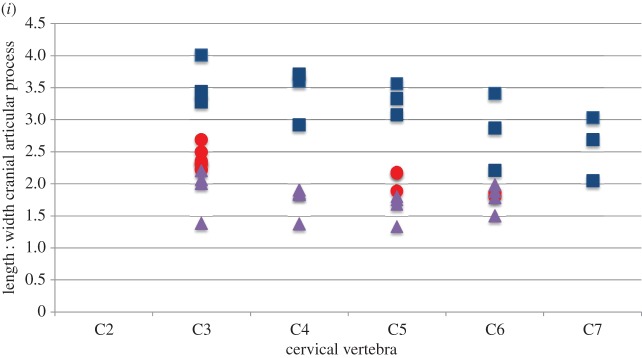
Scatter plots representing dimensions and characters comparing vertebral specimens of *Giraffa camelopardalis* (blue squares), *Samotherium major* (red circles) and *Okapia johnstoni* (purple triangles). Each variable is represented as a ratio between two measurements, or as an angle so as to eliminate body size differences between the three taxa. (*a*) Centrum length : minimum width, (*b*) anterior width : minimum width, (*c*) centrum length : height of spinous process, (*d*) length of spinous process : length of spinous process at base, (*e*) angle of spinous process, (*f*) angle of ventral tubercle, (*g*) angle of dorsal tubercle, (*h*) length : width of cranial articular facet and (*i*) length : width of cranial articular process.

Statistical analysis of the cervical vertebrae demonstrates that the *Samotherium* vertebrae share characters with both extant taxa,and are overall slightly closer to *G. camelopardalis*. Measurements used to compare the three taxa are represented in tables 1–6. Analysis of the more cranial (C2–C3) vertebrae demonstrates that *Samotherium* shares individual characters with either *G. camelopardalis* or *O. johnstoni*, and the more caudal (C5–C7) vertebrae appear intermediate between *G. camelopardalis* and *O. johnstoni* (table 7). The *Samotherium* length-to-width ratios plot between the two taxa, and appear closer to *O. johnstoni* (figure 6*a*). *Samotherium* also appears intermediate in several ratios, including anterior width : width, length : height of spinous process, cranial articular process and cranial articular facet (figure 6).

**Table 1.** Selected measurements of *Giraffa camelopardalis* C1–C7 vertebrae.

specimen no.	vertebra	centrum length	maximum length	minimum width	anterior width	posterior width	spinous process height	length of spinous process at base	length of spinous process
AMNH 27666	C1	139.58	169.14	82.23					
AMNH 27752	C1	144.8	173.01	105.03					
AMNH 82001	C1	150.56	184.61	101.06					
AMNH 27666	C2	314	330	34.26	119.76	76.17			
AMNH 27752	C2	309	323	51.49	117.14	87.34			
AMNH 82001	C2	297.99	326.37	31.86	114.58	61.57			
AMNH 27666	C3	308	337	37.63	83.74	80.63	46.14	233	175.25
AMNH 27752	C3	295	353	45.64	87.36	90.01	53.59	209.24	117.19
AMNH 82001	C3	310	350	34.11	64.53	72.52	51.49	230.68	164.25
AMNH 27666	C4	306	345	35.88	87.13	91.38	41.45	242.71	209.28
AMNH 27752	C4	310	347	44.74	90.7	101.83	44.47	261.01	186.43
AMNH 82001	C4	297	342	38.32	74.94	80.38	48.29	216.69	157.89
AMNH 27666	C5	292	336	41.11	101.65	102.27	54.28	247.7	197.82
AMNH 27752	C5	297	347	48.82	105.43	117.37	55.73	244.26	203.23
AMNH 82001	C5	293	341	43.33	90.75	94.08	50.92	230.35	163.83
AMNH 27666	C6	280	304	49.55	109.13	106.33	62.78	214.57	174.81
AMNH 27752	C6	292	316	58.36	121.51	118.45	56.82	196.24	164.69
AMNH 82001	C6	284	315	49.3	101.01	105.1	59.93	218.2	149.48
AMNH 27666	C7	252	230	62.12	114.23	132.3	114.22	103.99	88.16
AMNH 27752	C7	260.61	240.19	66.44	126.18	129.45	108.11	98.4	91.69
AMNH 82001	C7	271.79	236.43	68.26	109.98	122.25	96.32	89.61	59.61

**Table 2.** Selected measurements of *Okapia johnstoni* C1–C7 vertebrae.

specimen no.	vertebra	centrum length	maximum length	minimum width	anterior width	posterior width	spinous process height	length of spinous process at base	length of spinous process
AMNH 51197	C1	68.12	90.49	75.83					
AMNH 51198	C1	68.83	96.55	89.15					
AMNH 51213	C1	68.1	93.23	98.16					
AMNH 51197	C2	98	121.22	41.18	73.7	53.28			
AMNH 51198	C2	98.63	118.02	30.66	76.17	58.14			
AMNH 51213	C2	100.29	129.34	40.6	76.84	57.62			
AMNH 51197	C3	98.8	105.51	43.18	59.09	62.7	41.69	61.86	45.93
AMNH 51198	C3	99.65	104.07	43.49	64.14	63.26	33.86	52.13	38.77
AMNH 51213	C3	102.45	106.68	52.81	58.7	69.27	29.86	56.38	40.27
AMNH 51197	C4	103.03	106.73	58.92	67.92	73.35	39.55	55.39	43.16
AMNH 51198	C4	100.66	106.07	60.03	69.25	73.07	34.81	49.02	34.02
AMNH 51213	C4	109.16	102.83	65.05	69.11	82.18	35.1	44.71	34.78
AMNH 51197	C5	107.97	102.2	66.96	72.39	80.04	48.07	50.42	33.56
AMNH 51198	C5	101.07	100.43	68.71	74.73	78.31	41.3	41.31	28.28
AMNH 51213	C5	107.07	101.9	70.07	80.04	86.31	42.46	40.73	27.22
AMNH 51197	C6	107.4	92.13	67.22	80.15	79.56	60.42	42.58	32.3
AMNH 51198	C6	103.36	93.72	70.88	80.35	84.86	48.9	41.47	26.94
AMNH 51213	C6	111.02	97.91	73.24	82.65	84.98	45.96	40.85	28.03
AMNH 51197	C7	92.54	89.98	64.73	82.57	76.08	85.18	40.66	30.23
AMNH 51198	C7	87.26	86.3	64.09	87.78	81.78	89.7	42.98	35.82
AMNH 51213	C7	83.17	94.6	68.64	87.19	75.26	84.79	47.34	33.45

**Table 3.** Selected measurements of *Samotherium major* C1–C7 vertebrae.

specimen no.	vertebra	centrum length	maximum length	minimum width	anterior width	posterior width	spinous process height	length of spinous process at base	length of spinous process
PIM 429	C1	109.09		107.12					
PIM 430	C2	145.28	166.06	36.67	103.36				
PIM 1000	C2			44.5	109.57				
PIM 1001	C2	141.27	186.04	40.18	103.83				
PIM 432	C3	184.04		64.46	107.94	85.61	27.41	64.75	48.08
PIM 1012	C3	124.74	133.49	59.06			16.23	50.16	26.47
PIM 1002	C3	158.81	205.21	59.44	104.04	79.01	19.38	63.57	36.98
PIM 431	C3	181.55	234	63.55	91.71	85.47	18.8	64.43	24.63
PIM 1005	C3	157.53		54.87	90.59			50.37	28.37
PIM 1011	C3	139.65		58.91	97.42		20.44	61.17	34.55
PIM 433	C5	178.25	181.76	69.27	98.85	98.79		36	30
PIM 1006	C5	162.08		56.34				33.46	19.28
PIM 1003	C5	154.6	173.55	79.56	114.56	106.91	41.52	58.64	49.35
PIM 435	C6	171.42	179.65	72.1	104.65		50.47	73.79	44.82
PIM 434	C6	144.84	170.48	85.23	119.85	99.89	60.35	79.07	51.27
PIM 436	C7	143.65	158.55	85.78	127.17	107.59	60.69	70.05	60.98

**Table 4.** Characters used to evaluate *Giraffa camelopardalis* C2–C7 vertebrae.

specimen no.	vertebra	angle of spinous process	angle of ventral tubercle	angle of dorsal tubercle	L : W of cranial articular facet	L : W of cranial articular process
AMNH 27666	C2	164		8		
AMNH 27752	C2	162		10		
AMNH 82001	C2	160				
AMNH 27666	C3	110	82	11	1.9	4.01
AMNH 27752	C3	107	80	12	1.52	3.28
AMNH 82001	C3	104	78	11	1.87	3.44
AMNH 27666	C4	117	88	11	1.9	3.6
AMNH 27752	C4	98	85	16	1.58	3.71
AMNH 82001	C4	93	84	12	1.59	2.92
AMNH 27666	C5	92	87	15	1.77	3.56
AMNH 27752	C5	90	85	22	1.33	3.33
AMNH 82001	C5	93	86	15	1.33	3.08
AMNH 27666	C6	81	81	18	1.84	3.41
AMNH 27752	C6	62	50	24	1.37	2.21
AMNH 82001	C6	95	72	16	1.62	2.87
AMNH 27666	C7	67	83	20	2.14	3.03
AMNH 27752	C7	63	81	25	1.72	2.05
AMNH 82001	C7	63	69	22	1.55	2.69

**Table 5.** Characters used to evaluate *Okapia johnstoni* C2–C7 vertebrae.

specimen no.	vertebra	angle of spinous process	angle of ventral tubercle	angle of dorsal tubercle	L : W of cranial articular facet	L : W of cranial articular process
AMNH 51197	C2	112		20		
AMNH 51198	C2	130		20		
AMNH 51213	C2	131		23		
NMNH 399337	C2	105		19		
AMNH 51197	C3	79	49	28	0.98	1.39
AMNH 51198	C3	77	55	27	1.14	2.01
AMNH 51213	C3	69	37	22	1.11	2.21
NMNH 399337	C3	72	51	18		2.07
AMNH 51197	C4	72	66	35	1.3	1.38
AMNH 51198	C4	65	58	18	1.1	1.91
AMNH 51213	C4	75	44	25	0.88	1.84
NMNH 399337	C4	68	55	16		1.86
AMNH 51197	C5	67	76	35	1.52	1.34
AMNH 51198	C5	66	58	30	0.84	1.76
AMNH 51213	C5	66	45	26	0.69	1.69
NMNH 399337	C5	61	53	23		1.81
AMNH 51197	C6	58		30	0.85	1.51
AMNH 51198	C6	57		25	1	1.99
AMNH 51213	C6	54		19	1	1.89
NMNH 399337	C6	55		14		1.79
AMNH 51197	C7	66		11	1.38	
AMNH 51198	C7	60			1.19	
AMNH 51213	C7	59		24	1.22	
NMNH 399337	C7	85		14

**Table 6.** Characters used to evaluate *Samotherium major* C2–C7 vertebrae.

specimen no.	vertebra	angle of spinous process	angle of ventral tubercle	angle of dorsal tubercle	L : W of cranial articular facet	L : W of cranial articular process
PIM 430	C2	55		20		
PIM 1000	C2					
PIM 1001	C2					
PIM 432	C3	68	34	9	1.36	2.5
PIM 1012	C3	77	60	12		2.33
PIM 1002	C3	43	60	10	1.26	2.32
PIM 431	C3	68	26	9	1.27	2.69
PIM 1005	C3	47	34	16	1.52	2.22
PIM 1011	C3	55		12	1.07	2.36
PIM 433	C5	70	27	19	1.37	2.18
PIM 1006	C5	67	50	12		2.17
PIM 1003	C5	54		14	1.16	1.89
PIM 435	C6	56	60	6	1.44	1.87
PIM 434	C6	64	64	17	1.54	1.81
PIM 436	C7	85		15	1.55

**Table 7.** *P*-values of ANOVA tests with post hoc analysis comparing various cervical vertebral characters of *Giraffa camelopardalis*, *Okapia johnstoni* and *Samotherium major*. Statistical significance is set at the 0.0028 level due to Bonferroni correction, and statistically significant values are in bold. L, length; Min, minimum; W, width; H, height; SP, spinous process; VT, ventral tubercle; DT, dorsal tubercle; O.j., *Okapia johnstoni*; G.c., *Giraffa camelopardalis*; S.m., *Samotherium major*.

	centrum L: Min W	anterior W: Min W	centrum L: H SP	L SP : L SP at base	angle SP	angle VT	angle DT	L : W cranial articular facet	L : W cranial articular process
**C2–C3**									
ANOVA	**<0.001**	0.060	**<0.001**	0.135	**<0.001**	0.008	**<0.001**	**0.002**	**<0.001**
*G*.*c*. versus *O*.*j*.	**<0.001**	*	0.003	*	0.024	*	**<0.001**	**0.001**	**<0.001**
*G*.*c*. versus *S*.*m*.	**<0.001**	*	0.034	*	**<0.001**	*	0.233	0.004	**<0.001**
*O*.*j*. versus *S*.*m*.	0.353	*	**<0.001**	*	0.008	**<0.001**	*	0.102	0.032
**C5–C7**									
ANOVA	**<0.001**	**<0.001**	**<0.001**	0.181	**0.001**	0.015	0.014	**0.001**	**<0.001**
*G*.*c*. versus *O*.*j*.	**<0.001**	**<0.001**	**<0.001**	*	**0.002**	*	*	**<0.001**	**<0.001**
*G*.*c*. versus *S*.*m*.	**<0.001**	**<0.001**	0.054	*	0.042	*	*	0.138	**0.001**
*O*.*j*. versus *S*.*m*.	0.094	0.012	0.084	*	0.437	*	*	0.027	0.240

#### 3.2.1 Morphological similarities between *Samotherium major* and *Giraffa camelopardalis*

**Figure 7.**
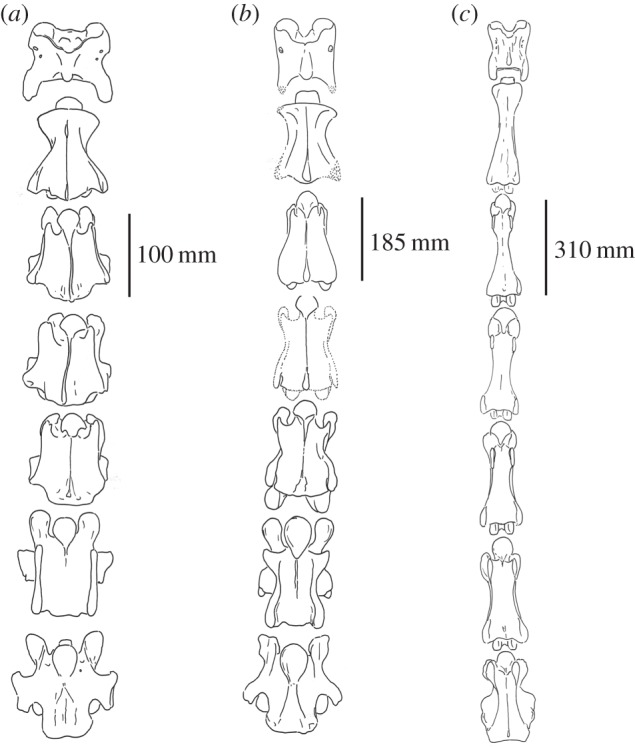
Ventral view of the C1–C7 vertebrae of (*a*) *Okapia johnstoni* (AMNH 51197), (*b*) *Samotherium major* and (*c*) *Giraffa camelopardalis* (AMNH 82001). The *S. major* vertebrae depicted are the most complete and undistorted specimens and are represented in figures 1–4.

*Atlas.* The cranial margin of the ventral arch is U-shaped, unlike *G. camelopardalis* where this edge is narrow and V-shaped (figure 7). The ventral tubercle protrudes caudal to the ventral arch and is within the arch in *G. camelopardalis*.

*Axis*. The ventral ridge is prominent, whereas it is faint and discontinuous in *G. camelopardalis* (figure 7).

**Figure 8.**
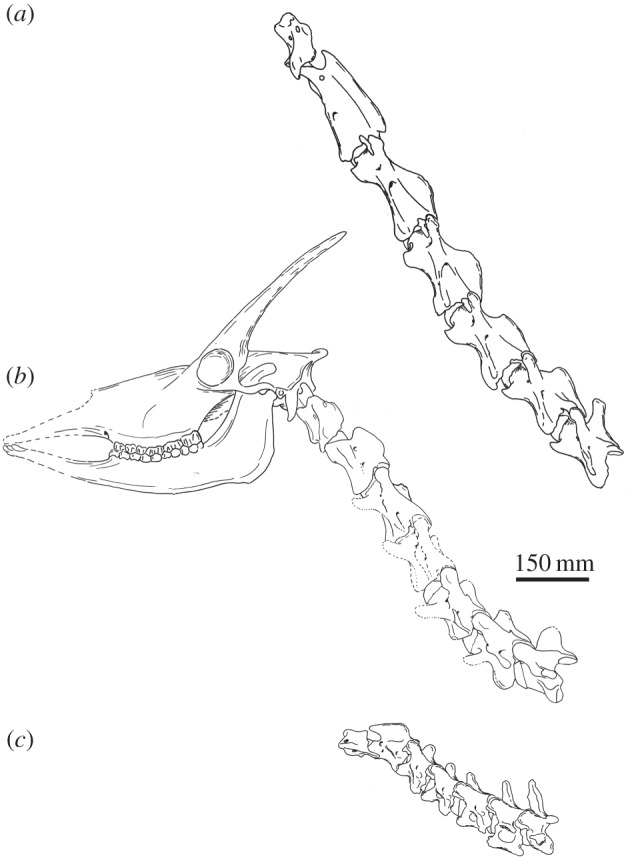
Lateral view of articulated C1–C7 vertebrae of (*a*) *Giraffa camelopardalis* (AMNH 82001), (*b*) *Samotherium major* and (*c*) *Okapia johnstoni* (AMNH 51197). A representative skull of *Samotherium major* using undistorted surfaces of several specimens (NHMBa29, NHMBa30, AMNH22808A, MGL11, SMNS 13285) is included to demonstrate verticality and a more complete illustration of this key extinct giraffid. The *S. major* vertebrae depicted are the most complete and undistorted specimens and are represented in figures 1–4.

*C3*. The thickest portion of the spinous process is positioned cranially, and it is caudal in *G. camelopardalis.* The caudal articular facets are circular and expansive, whereas they are more confined in *G. camelopardalis*. The pars interarticularis forms a well-defined separation between the dorsal lamina and the vertebral body, whereas this structure is faint in *G. camelopardalis* (figure 8). The caudal margin of the ventral vertebral body is laterally notched, unlike *G. camelopardalis*, where this region forms a transverse edge. There is a prominent ventral ridge on the ventral vertebral body, which is virtually absent in *G. camelopardalis* (figure 7).

*C5*. The spinous process is oriented and positioned more cranially, and it is central/caudal in *G. camelopardalis*. The caudal articular facets are expansive and rounded, whereas they are confined in *G. camelopardalis*. The transverse process protrudes laterally, unlike *G. camelopardalis* where it presents as an elongated ridge. The ventral tubercle is oriented cranially and is oriented ventrally in *G. camelopardalis* (figure 8). The caudal margin of the ventral vertebral body is laterally notched, unlike *G. camelopardalis*, where this region forms a transverse edge. The ventral ridge is prominent and expands laterally at the caudal margin of the ventral vertebral body, whereas it is absent in *G. camelopardalis* (figure 7).

*C6*. The spinous process is thickest cranially, unlike *G. camelopardalis* where the thickness is central. The spinous process is dorsally inclined, and it is cranially inclined in *G. camelopardalis*.

*C7*. The tip of the spinous process is rounded, whereas the tip is flat in *G. camelopardalis*. The transverse process is horizontally oriented, and it is inclined caudally in *G. camelopardalis*. There is no intertubercular plate, unlike *G. camelopardalis*, where this structure is present. The ventral extension is large and rounded, whereas it is pointed in *G. camelopardalis*. The ventral tubercle is absent, unlike *G. camelopardalis*, where the structure is present. There is a broad, central ventral ridge, which expands on the caudal half of the vertebra into two lateral ridges and a median ridge, whereas in *G. camelopardalis* there is a thin, continuous ventral ridge (figure 7).

#### 3.2.2 Morphological similarities between *Samotherium major* and *Okapia johnstoni*

*Atlas*. The cranial edge of the dorsal arch forms a U-shaped notch, whereas this forms a straight edge in *O. johnstoni*. The alar wings in lateral view are sigmoid shaped and are straight in *O. johnstoni*. *Okapia johnstoni* possesses a cranial protrusion on the lateral alar wings, which is absent in the other two taxa.

*Axis*. The cranial articular process is thick, whereas it is notably thinner in *O. johnstoni*. The cranial edge of the dens is flat with a slight central concavity, whereas it is somewhat pointed in *O. johnstoni*.

*C3*. The apex of the spinous process is inclined caudally, versus the cranial inclination in *O. johnstoni*. The cranial articular facets are oval shaped, unlike the rounded facets of *O. johnstoni*. The cranial articular process is long and slender, whereas it is shorter and less prominent in *O. johnstoni* (figure 8). The transverse process does not protrude as laterally as in *O. johnstoni*. The anterior arch, which defines the ridged connection between the cranial articular process and the ventral tubercle, is interrupted with the cranial articular process displaced caudally, whereas this arch is continuous in *O. johnstoni*. The ventral cranial bulge has a large, elongated, ventral extension onto the ventral vertebral body; the ventral extension is smaller and pointed in *O. johnstoni* (figure 7).

*C5*. The cranial articular facets are oriented dorsomedially, whereas they are dorsally oriented in *O. johnstoni*. The cranial articular process is long as slender, and it is shorter and less prominent in *O. johnstoni*. In lateral view, the pars interarticularis is flat, whereas it is sigmoid shaped in *O. johnstoni*. The anterior arch is interrupted, whereas it is continuous in *O. johnstoni* (figure 8).

*C6*. The cranial articular facets are oval shaped, unlike the rounded facets of *O. johnstoni*. There is a narrow separation between the caudal articular facets; this separation is wider in *O. johnstoni*. The anterior arch is interrupted, whereas it is continuous in *O. johnstoni*. The ventral vertebral body is hourglass shaped, whereas it is rectangular shaped in*O. johnstoni*. The ventral extension is extensive and is minimal in *O. johnstoni* (figure 7).

*C7*. The cranial articular process is elongated, unlike in *O. johnstoni*, where the cranial articular facets are approximated with the dorsal lamina.

#### 3.2.3 *Samotherium major* features intermediate between *Okapia johnstoni* and *Giraffa camelopardalis*

*Axis*. The thickness of the spinous process is intermediate between the large bulge exhibited in *O. johnstoni* and the confined expansion in *G. camelopardalis*. The spinous process is inclined caudally; it is less steep than that of *O. johnstoni,* but not horizontal as in *G. camelopardalis* (figure 8).

*C5*. The ventral extension is reduced in *S. major*; this structure is intermediate between the rounded ventral extension seen in *O. johnstoni*, and the pointed ventral extension exhibited in *G. camelopardalis* (figure 7).

*C6*. The length of the spinous process is intermediate between the elongated protrusion in *O. johnstoni* and the shortened state in *G. camelopardalis*. The transverse process is intermediate between the wide lateral protrusion seen in *O. johnstoni* and that of *G. camelopardalis* which is not visible in dorsal view. The position of the cranial opening of the foramen transversarium is between that of *O. johnstoni*, which is adjacent to the cranial bulge, and of *G. camelopardalis*, where the foramen is displaced caudally. There is a notch on the ventral lamina; in *O. johnstoni* the ventral lamina is complete, and in *G. camelopardalis* the ventral lamina is completely excavated (figure 8). The ventral ridge is continuous on the ventral vertebral body in *O. johnstoni*, absent in *G. camelopardalis*, and is present only on the cranial half of the vertebra in *S. major* (figure 7).

*C7*. The separation between the caudal articular facets in dorsal view resembles that of *O. johnstoni* in width, but is V-shaped like in *G. camelopardalis*.

## 4. Discussion

Cervical vertebral specimens are uncommon in fossil collections, both of giraffids and in general. Skulls and teeth have been historically favoured by many collectors and excavators [12]. The occurrence of an almost complete neck of an extinct giraffid is therefore exceptional, and allows for a comprehensive morphologic analysis. Moreover, the presently described specimens are relatively undistorted, and mainly intact. While isolated cervical vertebrae have been found at Pikermi, Thessaloniki, Sinap, Maragheh and Shanxi, this study demonstrates the first relatively complete neck specimen. Previous studies have focused on the cervicals of extinct giraffids, and used the individual vertebrae to discuss the evolution of neck elongation [1,8]. While single vertebrae are useful, and provide details about the anatomy, the entire cervical column in its complexity demonstrates morphological patterns and serial characteristics which are necessary to understand the dimensions and anatomy of extinct giraffid necks.

The giraffe neck is a favourable topic to science, as it exemplifies evolution with its specialized morphology. Giraffidae is an ideal family to examine cervical vertebrae. The two extant members represent short- and long-necked ruminants, and now the extinct *S*. *major* demonstrates an intermediate neck, thus representing a range of cervical lengths and presumably adaptations. Initially, Solounias [13] proposed that *G. camelopardalis* possesses an extra vertebra (totalling eight cervical) based on similarities of the typical ruminant C7 to the T1 of *G. camelopardalis* [13]. However, it is more likely that the similarities between the giraffe T1 and typical C7 are due to morphological transposition [14], as the phylogenetic constraints in mammals commonly prevent changes in the number of vertebrae [15]. Comparisons between the cervical columns of the two extant giraffid species reveal interesting differences in the serial morphologic patterns, where *G. camelopardalis* exhibits more homogenized vertebrae [2].

In observing features of extant artiodactyl cervical vertebrae, we find several characters that give insight into the verticality of the neck: the position of the dorsal tubercle of the atlas, the length of the C7 spinous process and the position of the median indentation of the palatine bone. Overall, in artiodactyls, there are three general positions of the neck at resting position. (i) The neck is relatively horizontal at resting position, where the atlas is approximately at the same level as C7, although the total neck is curved. This is exemplified by *Bos taurus* and *Syncerus caffer* [16]. (ii) The neck is semi-vertical, with the cervical vertebrae inclined at about 45° at resting position. This is the most common neck orientation in artiodactyls, represented by *O. johnstoni* and *Gazella* spp. [17]. (iii) The neck is relatively vertical, and the cervical vertebrae are inclined greater than 60° at resting position. *Giraffa camelopardalis, Camelus* spp. and *Litocranius walleri* exemplify this neck orientation [17,18].

We find that *S. major* exhibits cervical vertebral characters that more closely resemble those of the artiodactyls with the relatively vertical neck orientation. These taxa possess a centrally positioned dorsal tubercle of the atlas. Owing to rotation of the neck to achieve a more vertical position, the dorsal tubercle presumably becomes caudally displaced on the atlas. In *S. major*, the C1 dorsal tubercle is centrally positioned on the dorsal arch. Moreover, species exhibiting vertical necks often possess short C7 spinous processes relative to the vertebral body, as seen in *G. camelopardalis, Camelus* spp. and *Litocranius walleri* [2,17,18]. We believe this relates to the fibres of the laminar portion of the nuchal ligament attaching from T1 to C7. As expected, in *S. major* the spinous process of C7 is short (table 3). Additionally, the position of the soft palate relates to the orientation of the pharynx and larynx [9]. The epiglottis of the larynx forms a seal with the soft palate; therefore, their positions are closely interrelated. In a vertical neck, the vertebrae are oriented closer to the pharynx and larynx, which are consequently displaced anteriorly [9]. This displacement is reflected by a rostrally positioned palatine indentation. It is possible that *S. major* had a more vertical neck orientation than the giraffe. In *S. major*, the palatine indentation is between M2 and M3, which is more extreme than in *G. camelopardalis*, where the indentation is at the protocone of M3 [9]. We hypothesize that these characters are osteological correlates for the verticality of the neck, and that *S. major* possessed relatively vertically oriented cervical vertebrae at resting position.

*Samotherium major* exhibits cervical vertebral features that are uncharacteristic of ruminants. The *S. major* cervicals, in lateral view, have a ‘wedged’ morphology, where the caudal end of the vertebra has more depth, and the cranial end is smaller. This is atypical of ruminants; normally, the vertebral body and pedicles are of relatively equal size throughout the length. Several muscles that originate in the thorax insert on the caudal aspect of the cervical vertebrae [19], which is more extensive in *S. major*. We believe this matches the verticality osteological features, and that the presumed verticality of this extinct giraffid was strongly reinforced by muscular connections between the thorax and neck.

*Samotherium* *major* exhibits several unique morphologic features that are absent in the *G. camelopardalis* and *O. johnstoni* cervical vertebrae. In *S. major*, the cranial-most aspect of the spinous process of C2 is positioned caudally to the dens, whereas in *G. camelopardalis* and *O. johnstoni* it reaches the level of the dens. This increases the space between the atlas and axis, and would allow for increased dorsally directed motion of the atlas and the head. *Samotherium major* also has an atypical skull feature, where the occipital bone protrudes caudal to the skull (figure 8). We believe these two features are interrelated. Moreover, the dorsal lamina of *S. major* cervicals has a fossa, lateral to the spinous process. Ridges formed from the attachment of the multifidus muscle create this concavity. While *S. major* shares many features with the two extant giraffids, this extinct taxon also demonstrates characteristics atypical of giraffids, and ruminants in general.

The measured dimensions and observed morphological features of the *S. major* proximal vertebrae (C2–C3) demonstrate a mosaic of both *G. camelopardalis* and *O. johnstoni* qualities. The majority of measurements of these *S. major* vertebrae are statistically more similar to either *G. camelopardalis* or *O. johnstoni* (table 7). For example, the angle of the dorsal tubercle is most similar between *S. major* and *O. johnstoni*, whereas the angle of the spinous process of *S. major* more closely resembles *G. camelopardalis* (table 7). Similarly, the morphology of C2–C3 supports this observation that these vertebrae exhibit traits shared either with *G. camelopardalis* or with *O. johnstoni*. The cranial articular processes are more similar in shape to *G. camelopardalis*, and the caudal articular facets better match with *O. johnstoni*. It appears that the serial homology from one vertebra to another is complex, and individual morphological characters and measurements act independently within the same vertebra.

Features of the *S. major* distal (C5–C7) cervical vertebrae indicate a transitional state between the cervicals of *G. camelopardalis* and *O. johnstoni*. Measurements of these *S. major* vertebrae show few statistically significant differences with *G. camelopardalis* or *O. johnstoni*, indicating that the dimensions are between those of the two extant taxa. Morphologically, the C6 of *S. major* is exceptionally unique, and this vertebra exhibits several features intermediate between *G. camelopardalis* and *O. johnstoni.* Notably, in *S. major*, the ventral ridge is strong cranially and is absent caudally. In *G. camelopardalis* the ventral ridge is absent, and in *O. johnstoni* the ridge is fully developed and continuous on the ventral vertebral body. Additionally, the cranial opening of the C6 foramen transversarium in *S. major* is located between the cranial position seen in *O. johnstoni* and the more caudal position seen in *G. camelopardalis*. Lastly, the C6 of *S. major* possesses a ventral laminar wall with a notch visible in lateral view. In *O. johnstoni* the typical ventral lamina of ruminants is present, whereas in *G. camelopardalis* this lamina is completely excavated [2]. The notch seen in *S. major* represents a partial excavation of the ventral lamina, a feature intermediate between the two extant giraffids.

The cervical vertebrae of *S. major* have been previously referred to as ‘intermediate’, however this terminology in neck evolution can have several meanings. Length measurements of the *Samotherium* vertebrae indicate that the neck of this taxon is intermediate between that of short- and long-necked ungulates [1]. Similarly, the limb and neck measurements of *Samotherium* have been described to exhibit ‘giraffe-like’ lengthening, but do not yet reach the dimensions of *G. camelopardalis* [20]. Solounias [9] considered the *Samotherium* neck to be intermediate within Giraffidae based on cervical lengths and anatomical features [9]. A recent study of the evolutionary elongation of the giraffe neck found two significant lengthening stages within the family: cranial vertebral and caudal vertebral elongation. *Samotherium* is intermediate in the evolutionary process, as it demonstrates cranial lengthening, but lacks the caudal elongation features responsible for the extremely long neck of the giraffe [8]. In this study, we find two meanings for the word ‘intermediate’ with regards to the *S. major* cervical vertebrae. The C2–C3 vertebrae of *S. major* are ‘intermediate’ by exhibiting a mosaic of features shared either with *G. camelopardalis* or with *O. johnstoni*. The C5–C7 *S. major* vertebrae are also ‘intermediate’ in that they possess characteristics transitional between those of the two extant giraffid taxa. Our findings show both quantitative and qualitative features, where *S. major* falls between a short- and long-necked giraffid. While the meaning is multifaceted, the neck of *S. major* is truly ‘intermediate’ in every sense of the word.

## 5. Conclusion

In analysing morphological features and cervical measurements, we find the neck of *S. major* to be intermediate between *O. johnstoni* and *G. camelopardalis*. In the extinct taxon, the cranial (C2–C3) vertebrae demonstrate a mosaic of characters and dimensions that closely resemble either the okapi or the giraffe. The caudal region of the neck (C5–C7) appears more transitional between the two extant taxa. The C6 of *S. major* is exceptionally unique in that it has a partially excavated ventral lamina, and a ventral ridge that is developed cranially and yet absent caudally. Both quantitatively and qualitatively, we find the *S. major* neck to be truly intermediate between the okapi and the giraffe.

## Supplementary Material

Description of measurements and characters used to evaluate cervical vertebral specimens

## Data accessibility

The measurements supporting this study are included in the body of this article.
