# Supplementary material for: The cervical anatomy of Samotherium, an intermediate-necked giraffid
Source: R Soc Open Sci. 2015 Nov 25;2(11):150521. doi: 10.1098/rsos.150521 (PMC4680625; doi:10.1098/rsos.150521)
Supplement: Description of measurements and characters used to evaluate cervical vertebral specimens [file rsos150521supp1.docx]

Description of measurements and characters used to evaluate cervical vertebral specimens (modified from Danowitz and Solounias 2015)

Centrum length: distance between the cranial bulge and the caudal-most point of the vertebral body

Maximum length: distance between the cranial and caudal articular facets

Minimum width: distance between the narrowest points on the dorsal lamina

Anterior width: distance between the lateral edge of the cranial articular facets in dorsal view

Posterior width: distance between the lateral edge of the caudal articular facets in dorsal view

Spinous process height: maximum height along the median plane of the spinous process

Length of spinous process at base: distance between the cranial-most and caudal-most aspects of the spinous process on the dorsal lamina

Length of spinous process: distance between the cranial-most and caudal-most aspects of the spinous process 10 mm above the dorsal lamina

Angle of spinous process: angle formed between a median line running through the spinous process and the anterior-posterior axis of the vertebral body

Angle of ventral tubercle: angle formed between a median line running through the ventral tubercle and the anterior-posterior axis of the vertebral body

Angle of dorsal tubercle: angle formed between a median line running through the dorsal tubercle and the anterior-posterior axis of the vertebral body

Length:width cranial articular facet: length of the cranial articular facet divided by the width of the cranial articular facet

Length:width cranial articular process: distance between the cranial opening of the foramen transversarium and the tip of the cranial articular facet divided by the minimal width of the cranial articular process
